# Monitoring Covid-19 Policy Interventions

**DOI:** 10.3389/fpubh.2020.00438

**Published:** 2020-08-26

**Authors:** Paolo Giudici, Emanuela Raffinetti

**Affiliations:** ^1^Department of Economics and Management, University of Pavia, Pavia, Italy; ^2^Department of Economics, Management and Quantitative Methods, University of Milan, Milan, Italy

**Keywords:** concentration curve, contagion growth, statistical models, reproduction rate number, health policy interventions

## Abstract

A very key point in the process of the Covid-19 contagion control is the introduction of effective policy measures, whose results have to be continuously monitored through accurate statistical analysis. To this aim we propose an innovative statistical tool, based on the Gini-Lorenz concentration approach, which can reveal how well a country is doing in reducing the growth of contagion, and its speed.

## 1. Introduction

Coronavirus disease (Covid-19) is a novel coronavirus which causes severe respiratory illness (1). The first cases of pneumonia cases of unknown etiology, later ascribable to the new Covid-19, arose at the end of December 2019 in Wuhan, the capital city of Hubei (China), and later in other Asian countries, such as Korea and Iran. On 21 February 2020, the first cases were recorded in Italy and from there on the contagion rapidly extended, to other European countries (especially Spain, United Kingdom, France, and Germany) and, later, to the whole world (including the United States, Russia, and Turkey).

As no specific vaccine is yet available, all governments attempted to control the spread of the pandemic phenomenon. Extensive health policy measures were implemented with the purpose of reducing the person-to-person transmission of the virus.

To be effective, policy measures need an effective continuous monitoring of their results. In this respect, recent studies on policy monitoring were addressed to the study of Covid-19, both from an epidemiological and a statistical view point [see e.g., (2)].

The contribution of this paper is in this latter direction. One quadrimester after the outbreak of the pandemic in China, and one quarter after its worldwide spread, it becomes important to compare the containment policies undertaken by the different governments, to learn which have been most effective, and draw lessons for the management of the subsequent phase, which may include a possible relaxation of the measures, and a more strict statistical monitoring of their results. We propose an innovative statistical tool which can assess the effectiveness of policy measures in the containment of the Covid-19 contagion growth over time. This because the most important effort during the outbreak has been the reduction of the number of infected people which, in turn, determine a reduction in the severely hospitalized patients and, ultimately, a reduction of deaths.

The proposed tool has the purpose to detect the countries which achieved the best results in terms of reduction in number of contagions in the smallest time interval. An accurate analysis of the Covid-19 dynamics along the weeks can provide useful information to improve health policies and reorganize the related services. It is also very useful to plan future interventions, in case of new contagion outbursts.

The paper is organized as follows: Section 2 is devoted to the illustration of our proposal; Section 3 reports the results based on data concerning the Covid-19 cases detected in periods of about 2 months (9 weeks) in the time span between 20 January 2020 and 22 March 2020 in China and between 24 February 2020 and 26 April 2020 in Italy, Germany, Korea and USA; Section 4 concludes the paper with final comments.

## 2. Proposal

Most epidemiologic models, including the well-known Susceptible-Infected-Recovered (SIR) model [see e.g., (3, 4)] rely on the assumption that contagion counts *Y* can be well-explained by a function of *X*, (time) such as a linear, exponential, or logistic, indicating different growth patterns. To understand which function of *X* best fits *Y*, quantitative concordance measures taking time into account are needed. We propose to employ a method which uses a rank-based quantitative measure, extending what proposed in the predictive accuracy context by Giudici and Raffinetti (5) and Agosto et al. (6).

Let *P*_*c*_ = {*p*_*c*_1__, …, *p*_*c*_*n*__} denote the positive cases of Covid-19 and *D* the day of the occurred contagion, such that *D* = {1, …, *n*}. We can then build a curve *C*, according to Agosto et al. (6), as follows:

- re-order the *P*_*c*_ variable values by the ranks of variable *D* and denote them with *p*_*c*_*r*(_*d*_*i*__)__, where *i* = 1, …, *n* and *r*(·) represents the rank;
- determine the curve *C* coordinates, i.e., $\left(i/n,\left(1/\left(n\overset{-}{p_{c}}\right)\right)\overset{i}{\underset{j=1}{\sum }}p_{c_{r\left(d_{j}\right)}}\right)$, where $\overset{-}{p_{c}}=\frac{1}{n}\overset{n}{\underset{i=1}{\sum }}p_{c_{i}}$ and *p*_*c*_*r*(_*d*_*j*__)__ is the *j*-*th* *P*_*c*_ variable value ordered by the rank of the corresponding *d*_*j*_ value (with *j* = 1, …, *i*);
- provide the set of the linear curve points of coordinates (*i*/*n, i*/*n*).

The *C* curve is a *concordance curve* since it measures the concordance between the ranks of the *P*_*c*_ variable values *r*(*p*_*c*_*i*__) and the ranks of the *D* variable *r*(*d*_*i*_), for *i* = 1, …, *n*. Based on the *C* curve behavior, five main scenarios may arise: (a) a perfect concordant relationship between the Covid-19 positive cases *P*_*c*_ and time *D*, which occurs iff *r*(*p*_*c*_*i*__) = *r*(*d*_*i*_) for any *i* = 1, …, *n*; (b) a perfect discordant relationship between the Covid-19 positive cases *P*_*c*_ and time *D*, which occurs iff *r*(*p*_*c*_*n*+1−*i*__) = *r*(*d*_*i*_) for any *i* = 1, …, *n*; (c), (d) a partial discordant and then concordant relationship or a partial concordant and then discordant relationship between the Covid-19 positive cases *P*_*c*_ and time *D*, which occur iff the *P*_*c*_ variable ranks are partly discordant and partly concordant with the *D* variable ranks; (e) a uniform relationship between the Covid-19 positive cases *P*_*c*_ and time *D*, which occurs iff the number of Covid-19 positive cases uniformly increases over time, i.e., *p*_*c*_*i*__ = *p*_*c*_*j*__ for any *i* = 1, …, *n* and *j* = 1, …, *n*, so that $p_{c_{i}}=p_{c_{j}}=\overset{-}{p_{c}}$, being $\overset{-}{p_{c}}$ the mean of positive Covid-19 cases.

**Figure 1:**
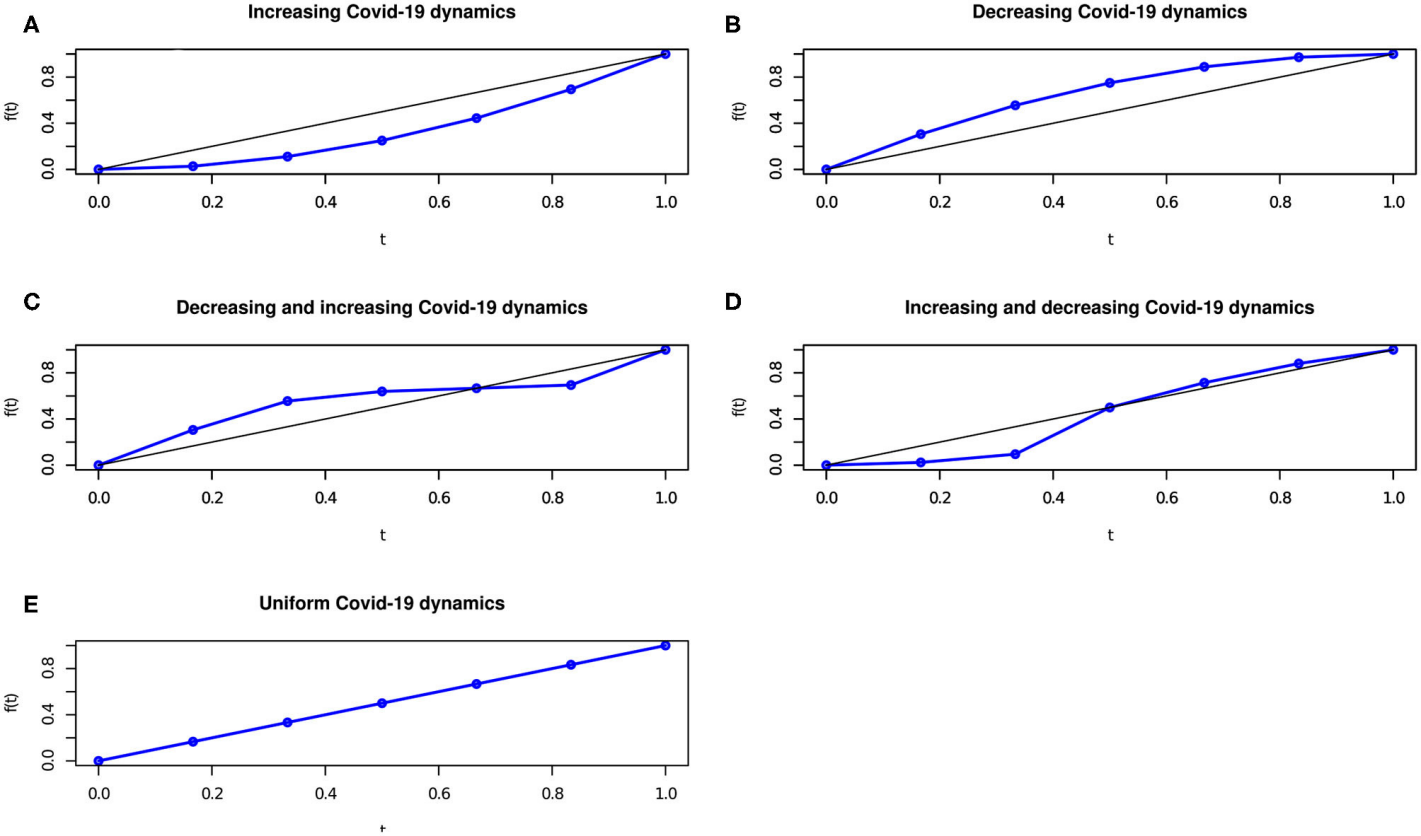
The Covid-19 dynamics behavior in scenarios **(A–E)** in terms of concordance curve.

As an example, the graphical representation of the *C* concordance (in blue) curve and the linear (in black) curve, corresponding to the bisector curve of the unit side square, is reported in Figure 1.

Figures 1A–E display the Covid-19 spread over time, in the cases where *r*(*p*_*c*_*i*__) = *r*(*d*_*i*_) for any *i* = 1, …, *n*; *r*(*p*_*c*_*n*+1−*i*__) = *r*(*d*_*i*_) for any *i* = 1, …, *n*; *r*(*p*_*c*_*n*+1−*i*__) = *r*(*d*_*i*_) and *r*(*p*_*c*_*i*__) = *r*(*d*_*i*_) for some *i* = 1, …, *n*; *r*(*p*_*c*_*i*__) = *r*(*d*_*i*_) and *r*(*p*_*c*_*n*+1−*i*__) = *r*(*d*_*i*_) for some *i* = 1, …, *n*; $r\left(p_{c_{i}}\right)=r\left(\overset{-}{p_{c}}\right)=r\left(d_{i}\right)$ for any *i* = 1, …, *n*.

To have a picture of the Covid-19 spread, daily contagions (variable *P*_*c*_) can be re-ordered by time (variable *D*) to show if the number of contagions increases, decreases or remains stable over time. Specifically, if the concordance curve is below the bisector curve, the number of contagions increase with time whereas if the concordance curve is above the bisector curve, the number of contagions reduces with time.

Due to its features, the concordance curve can be exploited to summarize the “distance” between the *P*_*c*_ and the *D* values, in terms of the “discrepancy” between their corresponding ranks. A summary index, pointed out with *RG* (acronym of Rank Graduation), can be introduced as

(1) $$\begin{array}{l} RG=\overset{n}{\underset{i=1}{\sum }}\frac{\left\{\left(1/\left(n{\overset{-}{p}}_{c}\right)\right)\sum_{j=1}^{i}p_{c_{r\left(d_{j}\right)}}-i/n\right\}}{i/n}=\overset{n}{\underset{i=1}{\sum }}\frac{\left\{C\left(p_{c_{r\left(d_{j}\right)}}\right)-i/n\right\}}{i/n}, \end{array}$$

where $C\left(p_{c_{r\left(d_{j}\right)}}\right)=\frac{\overset{i}{\underset{j=1}{\sum }}p_{c_{r\left(d_{j}\right)}}}{\overset{n}{\underset{i=1}{\sum }}p_{c_{i}}}$ is the cumulative of the (normalized) *P*_*c*_ variable values.

Note that the measure in Equation (1) is similar to that proposed in (6). The *RG* is equal to 0 in the case of a perfect overlap between the *C* concordance curve and the bisector curve: this reflects that the epidemic is under control, with the number of cases increasing at a constant rate.

When the concordance curve is below the bisector curve, the number of contagions increases with time, leading to a negative *RG* value. When the concordance curve is above the bisector curve, the number of contagions reduces with time, leading to a positive *RG* value.

The dynamics of contagion may vary over time and an analysis of the *RG* trend in different time intervals may be useful to better understand the most problematic periods as well as the time in which a change in the increase or decrease of cases arises. As the overall *RG* measure is proportional to the area between the concordance and the bisector curves, the measure of the *RG* variation over time, associated with a specific time interval [*t*_*h*−1_, *t*_*h*_] with *h* = 1, …, *H*, can be determined multiplying the *RG* index by the area between the concordance and bisector curve corresponding to the time interval [*t*_*h*−1_, *t*_*h*_].

## 3. Results

In this section we apply the concordance curve and the associated summary *RG* measure to assess the Covid-19 dynamics in the most infected countries in the world. The analyzed data report the daily number of positives cases^1^ along a time interval of nine weeks (63 days), starting from day 24 February 2020 until day 26 April 2020. This time interval was taken into account for both European and non European countries, except for China, where the contagion already occurred in January 2020. To provide a coherent comparison of the Covid-19 spread, we focus on the first nine weeks of Covid-19 spread in China, corresponding to the time range between 20 January 2020 and 22 March 2020.

**Figure 2:**
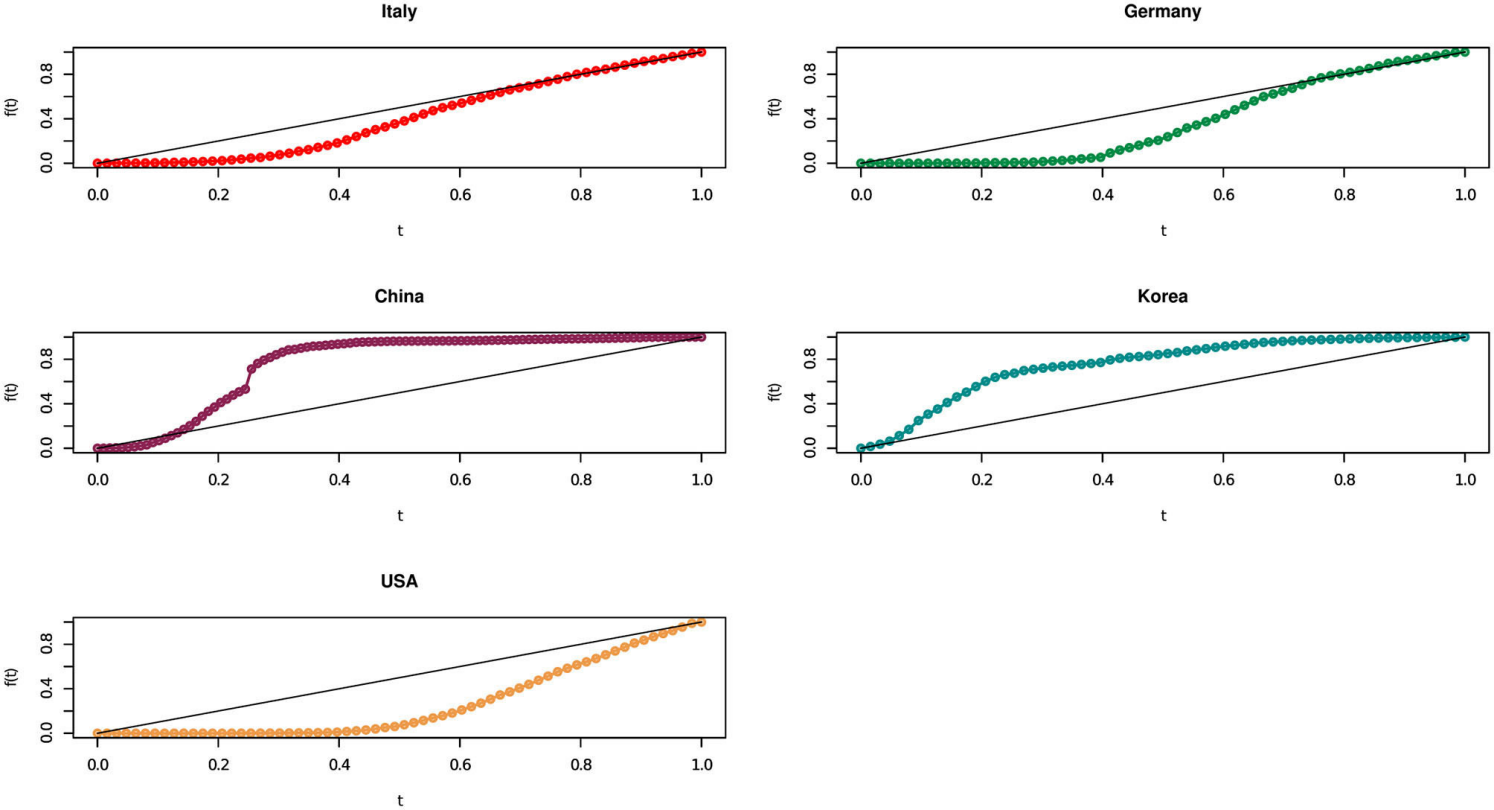
The concordance curve behavior for Italy, Germany, China, Korea, and USA.

Figure 2 presents the results of our methodology, in a graphical representation, for the considered European and non European countries.

We recall that the more the concordance curve in Figure 2 approaches the bisector curve, the more the growth of contagions become uniform over time. From Figure 2 it seems that Italy moved to a linear trend ahead than Germany. In other words, although Italy started with a very high number of cases, its policy containment measures have been quite effective in rapidly bringing down an exponential growth to a linear one.

We need a more thorough understanding of the concentration dynamics along time. A notable effect concerns Germany, whose area appears similar (or slightly higher) than Italy but which shows, in recent times, a growth that is less than linear (above the bisector curve), indicating that this country is doing quite well in containing the virus.

Moving to non European countries, Figure 2 clearly shows how, on the basis of the analyzed official data, China rapidly brought down contagion numbers: its concentration curve started with a strongly increasing Covid-19 pattern, rapidly followed by a reduction in the number of cases, along the nine weeks between 20 January 2020 and 22 March 2020. Even better, Korea in the 9 weeks between 24 February 2020 and 26 April 2020 has first a linear growth, which translate into a decreasing one, while the USA presents a concordance curve behavior always below the bisector curve.

We now move to the summary statistical representation of our results, by means of the proposed *RG* measure. Before looking at that, we present some context summary statistics, which indicates the incidence of the contagion and the incidence of tests, in the considered countries, as of July 2020. The incidence is calculated as the total number of observed cases (or of performed tests) divided by the country's population. Table 1 presents the results.

**Table 1:** Incidence by country and test rate.

**Country**	**Population**	**Covid-19 cases**	**Incidence (%)**	**Test rate (%)**
Italy	60,359,546	240,961	0.398	9.09
Germany	82,366,300	196,738	0.234	7.01
China	1,433,783,686	83,542	0.006	6.28
Korea	60,359,546	12,967	0.025	2.55
USA	329,311,764	2,854,976	0.862	10.75

From Table 1 note that, in population relative terms, the USA reports the highest incidence (at almost 0.9%), even though the virus outbreak was observed later, followed by Italy (around 0.4%), then Germany (around 0.2%), Korea (around 0.02%), and China (around 0.006%). The testing rate is quite in line with the incidence, with the USA first, followed by Italy, Germany, China and, finally, Korea.

We now compare countries in terms of the summary *RG* measure. Table 2 presents the overall *RG* measure for each country, indicating how fast the incidence observed in Table 1 has grown, and how fast it has been contained.

**Table 2:** *RG* value by country.

**Country**	***RG***
Italy	−24.56
Germany	−31.52
China	+10.07
Korea	+52.48
USA	−41.66

Table 2 clearly shows that Korea, followed by China, are the best performing countries: in both cases the curve has been below a linear growth trend for most of the time. In line with our comments to Figure 2 the two countries are followed by Italy and Germany, which managed to bring down their large numbers thanks to very severe containment policies (Italy) or extensive testing (Germany). Last, the curve of the USA shows a still persistent difficulty in pandemic control.

As mentioned several times, to compare policies, it is important to understand how the *RG* measure has evolved over time, in each country. The results of the *RG* variation over time are shown in Tables 3, 4.

**Table 3:** *RG* variation over time and *R*_0_ (China)—Week 1: 20 January 2020 to 26 January 2020; Week 2: 27 January 2020 to 2 February 2020; Week 3: 3 February 2020 to 9 February 2020; Week 4: 10 February 2020 to 16 February 2020; Week 5: 17 February 2020 to 23 February 2020; Week 6: 24 February 2020 to 1 March 2020; Week 7: 2 March 2020 to 8 March 2020; Week 8: 9 March 2020 to 15 March 2020; Week 9: 16 March 2020 to 22 March 2020.

**China**		
**Week**	**RG variation**	***R*_0_**
Week 1	−0.35	-
Week 2	−0.57	7.00
Week 3	+0.28	1.84
Week 4	+1.87	1.37
Week 5	+2.90	0.27
Week 6	+2.51	0.34
Week 7	+1.90	0.30
Week 8	+1.15	0.20
Week 9	+0.38	1.94
Overall *RG*	+10.07	

**Table 4:** *RG* variation over time and *R*_0_ (Italy, Germany, Korea and USA)—Week 1: 24 February 2020 to 1 March 2020; 2 March 2020 to 8 March 2020; Week 3: 9 March 2020 to 15 March 2020; Week 4: 16 March 2020 to 22 March 2020; Week 5: 23 March 2020 to 29 March 2020; Week 6: 30 March 2020 to 5 April 2020; Week 7: 6 April 2020 to 12 April 2020; Week 8: 13 April 2020 to 19 April 2020; Week 9: 20 April 2020 to 26 April 2020.

**Week**	**RG variation**	***R*_0_**
**Italy**		
Week 1	−1.61	-
Week 2	−4.54	4.53
Week 3	−6.47	3.21
Week 4	−6.31	2.12
Week 5	−3.89	1.20
Week 6	−1.67	0.83
Week 7	−0.47	0.86
Week 8	+0.21	0.86
Week 9	+0.19	0.82
Overall *RG*	−24.56	
**Germany**		
Week 1	−1.43	-
Week 2	−4.25	7.67
Week 3	−6.87	4.01
Week 4	−8.33	5.99
Week 5	−6.89	1.76
Week 6	−3.78	1.26
Week 7	−0.73	0.73
Week 8	+0.36	0.68
Week 9	+0.40	0.68
Overall *RG*	−31.52	
**Korea**		
Week 1	+1.25	-
Week 2	+6.62	1.08
Week 3	+8.88	0.30
Week 4	+8.13	0.80
Week 5	+7.36	0.84
Week 6	+6.54	0.99
Week 7	+5.17	0.40
Week 8	+3.25	0.54
Week 9	+1.11	0.45
Overall *RG*	+48.31	
**USA**		
Week 1	−1.09	-
Week 2	−3.27	10.71
Week 3	−5.43	6.92
Week 4	−7.42	9.45
Week 5	−8.37	4.11
Week 6	−7.50	1.92
Week 7	−5.14	1.16
Week 8	−2.77	0.94
Week 9	−0.67	0.84
Overall *RG*	−41.66	

From Table 3, note that China moved from a negative to a positive *RG* value already during the third week from the reported outbreak, highlighting that the contagion was contained rather promptly. Moreover, in the fifth week the *RG* reaches the highest value denoting the greatest decreasing reduction in number of Covid-19 positive cases over time.

Italy and Germany (Table 4) record positive *RG* values only during the latest two weeks. The presence of this *RG* positive value is due to the fact that the trend of contagion becomes stable overtime, indicating that the countries have reached a contagion peak. It is worth noting that the *RG* associated with Germany in the last week takes a greater value than that of Italy. This results is consistent with the fact that between the 56-th and 63-th days the Germany concordance curve starts lying slightly above the bisector curve. These findings indicate that Germany has been able to contain the contagion, and so has Italy, which however started before and had higher contagion counts.

Consider now the situation in non-European countries. From Table 4, note that Korea always reveals a positive *RG* value for the whole time-interval. More precisely, in the first days of the first week, the number of contagions uniformly increase while in the remaining time it follows a decreasing trend. This indicates a very effective containment policy, sustained by a high level of testing as shown in Table 1. On the other hand, USA do not record a reversal of the contagion trend overtime. This may indicate a late start but also a less effective containment policy.

To gain further insight into the advantages of our proposal, we present a comparison of the *RG* measure with the reproduction rate (number) *R*_0_. We recall that the reference epidemiologic model, the Susceptible Infected Recovered (SIR) methodology [see, for example, (2)] is essentially based on the determination of *R*_0_, calculated as:

(2) $$\begin{array}{l} R_{0}=\frac{b*\left(1-a\right)*E\left(T\right)}{h} \end{array}$$

where, for any individual in a population: *b* is the probability of becoming infected (infection rate); *E*(*T*) is the mean incubation time of the disease, in case of infection; *h* is the probability of detecting the infected case (confirmation rate); *a* is the probability of isolating the contacts of the infected case (quarantine rate). Using what available in the SIR modeling literature, Agosto and Giudici (2) proposes how to set these parameters to study a possible evolution of the Covid-19 outbreak: *T* is based on a Gamma distribution, with expected value equal to *E*(*T*) = 7.5, 1 − *a* is set equal to *h*, without loss of generality; and *b* is estimated from a statistical model: exponential (as in standard SIR models) or autoregressive [as in (2)]. Here we will follow a non parametric approach, according to which *b* can be calculated as the ratio between the new observed cases $\hat{\gamma_{t}}$ at *t* and the mean number of observed cases in the previous (*t* − *l*, …, *t* − 1) days. In line with the expected infection time (7.5 days for Covid-19), *l* is fixed equal to 7, so that:

(3) $$\begin{array}{l} \hat{b}=7*\frac{{\hat{\gamma }}_{t}}{\sum_{i=t-l}^{t-1}{\hat{\gamma }}_{i}}\text{ with: }i=1,\ldots ,t-1;l=1,\ldots ,7. \end{array}$$

Following the previous step, a baseline level of *R*_0_ can be calculated as follows:

(4) $$\begin{array}{l} R_{0}=E\left(T\right)*\hat{b} \end{array}$$

which, assuming *E*(*T*) = 7.5, gives $R_{0}=7.5*\hat{b}.$ Epidemiologically, the higher the *R*_0_ the higher the number of people that will be infected and, eventually, will be hospitalized in severe conditions, or will die. A value of *R*_0_ less than 1 indicates that the epidemic is under control, and is leading to an upper bound of cases. From our proposed definition of *b*, it is clear that policy making (and its compliance) can affect it by changing *a*, *h*, or both.

In our perspective, we refer to weekly intervals, leading formula of *R*_0_ becoming $R_{0}=\frac{{\hat{\gamma }}_{w}}{{\hat{\gamma }}_{w-1}}$, where ${\hat{\gamma }}_{w}$ and ${\hat{\gamma }}_{w-1}$ represent the total new Covid-19 positive cases observed in week *w* and in week *w* − 1, respectively. It follows that the value for *R*_0_ is not available for the first week.

The results of our weekly *R*_0_ are shown in Tables 3, 4, and can be compared with those of the *RG* values, for all weeks except the first. The overall trend of *R*_0_ confirms that of the *RG* statistics, with very low values for Korea, fast decreasing values for China, slowly decreasing values for Italy and Germany and even slower for the USA. However, the *R*_0_ appears more suitable to indicate “local” variations rather than to evaluate policies in a longer time horizon. This is shown, for example, in the case of China, in which Week 9 indicates a rebound of the *R*_0_, due to the emergence of a relatively small number of cases, but large with respect to the cases of the previous week. This does not indicate that the containment policy is failing but rather a “warning sign.”

## 4. Conclusions

We have presented a novel methodology that can be very helpful to summarize and compare the effectiveness of Covid-19 containment measures, in different countries. Specifically, we have applied our proposed measures to the most infected world countries, in order to assess if an increasing, uniform or decreasing relationship occurs between the number of positive Covid-19 cases and time.

Our empirical findings show that, starting from 13 April 2020, Italy and Germany have achieved at least a uniform or slightly decreasing trend of the contagion dynamics. With regard to the non-European countries, China and Korea appear as the most effective in containing the contagion, while USA do not perform well due to an evident spread in the number of contagions.

Future research may involve comparison of other components of the disease epidemiology, such as the number of severely hospitalized people, and the number of deaths.

## Data Availability Statement

Publicly available datasets were analyzed in this study. This data can be found here: https://www.who.int/emergencies/diseases/novel-coronavirus-2019/situation-reports.

## Author Contributions

This paper is the result of a close collaboration between the two authors. However, PG wrote sections Introduction and Conclusions and ER wrote sections Proposal and Results. Both authors contributed to the article and approved the submitted version.

## Conflict of Interest

The authors declare that the research was conducted in the absence of any commercial or financial relationships that could be construed as a potential conflict of interest.

## Footnotes

^1^Note that we do not resort to the standardized data (number of Covid-19 positive cases/number of population), since the proposed *RG* measure is invariant with respect to this kind of data transformation. Indeed, if the term *C*(*p*_*c*_*r*(_*d*__*j*_)__) in Equation (1) is divided by the number of population *n*_*pop*_, it results that $C\left(p_{c_{r\left(d_{j}\right)}}\right)=\frac{\overset{i}{\underset{j=1}{\sum }}\frac{p_{c_{r\left(d_{j}\right)}}}{n_{pop}}}{\overset{n}{\underset{i=1}{\sum }}\frac{p_{c_{i}}}{n_{pop}}}$ which exactly returns $\frac{\overset{i}{\underset{j=1}{\sum }}p_{c_{r\left(d_{j}\right)}}}{\overset{n}{\underset{i=1}{\sum }}p_{c_{i}}}$.

## Supplementary Material

All used data are publicly available at the WHO website. Data analysis was made using the R package, and the employed code is available upon request from the authors.
